# Hereditary Predisposition

**Published:** 1846-02

**Authors:** 


*Hereditary Predisposition.*—There is no subject connected with medicine which would more amply repay a scientific investigation than this. The facts are numerous, and can be arranged on an irrefragible general principle, namely: that every portion of the body of the offspring participates more or less in the physiological conditions of the parents. The first step in the inquiry would be, to ascertain the physiological laws of hereditary transmission. The true direction in which to seek this is in embryology, and particularly in the development of tissues. Dr. Siebert remarks, to illustrate our views, that the gout of the parents may appear in the offspring as stone, or disease of the heart. This we know to be true, and the explanation, we conceive, is, that the serous layer of embryo has received the hereditary predisposition of the parent. This predisposition is shown in a tendency to disease of the embryonic products of the serous layer, namely, the proper serous membranes and the bones and muscles with their appendages, namely, sero-synovial sacs, tendons, cartilages, &c. With this predisposition a mordid action in and a metamorphosis of the serous tissues is more frequent. Thus, in persons of this constitution, the muscular and tendinous fibres, and the cartilages. will change into bone—the serous membranes into cartilage—while the sub-serous membranes of the hollow muscles become the nidus of ossific deposits. Dr. Siebert remarks that gonorrheal fathers have scrofulous rachitic children. In this example, the mucous layer of the embryo and the mucous surfaces and tissues derived therefrom have the morbid predisposition of which the gonorrhea and the scrofula are special manifestations.

Special organs suffer also hereditarily, just as minute personal traits are transmitted to offspring. M. Boucher inquired into the fate of 58 individuals, the offspring of 14 epileptic females. Of these 32 died of convulsions in infancy, and of the 26 remaining, 7 had different nervous affections, 2 convulsions of an intermittent type, 2 were epileptics, and 1 hysterical; so that 14 only of 26 who grew up, remained free from disease of the nervous system. As we shall revert to this important question elsewhere, nothing more need here be said, except that Dr. Siebert's discussion of the subject is superficial and contains nothing novel.—*Brit. & For. Med. Rev.—Bul. Med. Sci.*

